# Diabetes Mellitus in Pancreatic Cancer Patients in the Czech Republic: Sex Differences

**DOI:** 10.1155/2012/414893

**Published:** 2012-06-27

**Authors:** Jan Trna, Petr Dítĕ, Arona Adamcová, Brianna J. Crawford, Markéta Hermanová

**Affiliations:** ^1^Department of Internal Medicine and Gastroenterology, University Hospital and Faculty of Medicine, Masaryk University Brno, Jihlavska 20 62500 Brno, Czech Republic; ^2^University of North Dakota, Grand Forks, ND 58202, USA; ^3^Department of Pathology, University Hospital and Faculty of Medicine, Masaryk University Brno, 62500 Brno, Czech Republic

## Abstract

*Aims*. The prevalence of diabetes mellitus in pancreatic cancer patients and control subjects was compared. *Methods*. Retrospective evaluation of 182 pancreatic cancer patients and 135 controls. The presence of diabetes was evaluated and the time period between the diagnosis of diabetes and pancreatic cancer was assessed. A subanalysis based on patient sex was conducted. *Results*. Diabetes mellitus was present in 64 patients (35.2%) in pancreatic cancer group and in 27 patients (20.0%) in control group (*χ* ^2^ = 8.709; *P* = 0.003). In 18 patients (28.1% of diabetic pancreatic cancer patients) diabetes was new-onset. Diabetes was new-onset in 23.3% of females compared to 38.1% of males (*χ* ^2^ = 1.537; *P* = 0.215). The overall prevalence of diabetes was significantly higher among female pancreatic cancer patients (25% versus 43.9%; *χ* ^2^ = 7.070, *P* = 0.008), while diabetes prevalence was equally represented in the control group patients (22.1% versus 17.2%; *χ* ^2^ = 0.484, *P* = 0.487). *Conclusion*. The prevalence of diabetes mellitus in study group of pancreatic cancer patients was significantly higher when compared to control group. Pancreatic cancer patients with diabetes were predominantly females, while diabetes was equally prevalent among sexes in the control group. Therefore, patient sex may play important role in the risk stratification.

## 1. Background

Pancreatic cancer (PC) represents the fourth leading cause of cancer death with an increasing incidence. Despite the efforts for the improvement of diagnostics and therapy, the prognosis of patients with PC remains dismal, with an overall 5-year survival rate of approximately 5% [1–3]. One of the primary causes for this unfavorable situation is the long asymptomatic course of the disease, so when the disease is discovered, it is usually in the advanced stage and curative surgery is typically impossible [4]. Invasiveness, early metastazing, and resistance to radio- and chemotherapy are additional reasons for the disappointing prognosis. 

In view of these facts, current pancreatologic research focuses on the identification of risk factors and the determination of high-risk patient groups to increase the potential for screening and early diagnosis of PC. Currently there are screening programs for patients with several genetic disorders, including hereditary chronic pancreatitis, who are at a higher risk of progression to PC [5]. But high risk groups in the general population are still not well defined. 

One of the long debated factors linked to pancreatic cancer is diabetes mellitus (DM). The association of DM and PC is complex and not completely understood. Whether DM is a risk factor for the development of PC or its first symptom remains a matter of research and debate [6–10]. However, subjects with new-onset DM have been shown to have a higher than expected likelihood of having PC [11, 12] and screening of new-onset diabetics has been proposed as a strategy to improve unfavorable outcomes of PC treatment [13].

The primary aim of our study was to retrospectively establish the prevalence of DM in the group of PC patients autopsied in our institution, to record the chronology of the diagnoses and compare the results with the control group.

## 2. Methods

We retrospectively evaluated autopsy reports and related documentation including final hospital release forms of 182 consecutive PC patients who died between 2001 and 2005 in the South Moravian region of the Czech Republic. The study was restricted to patients diagnosed with PC prior to their death based on clinical presentation, imaging methods, and/or results of surgery and autopsy with corresponding diagnosis in the Department of Pathology of University Hospital Brno. As a control group, 135 patients of comparable age and sex distribution who died of non pancreas-related disease during the same time interval were randomly selected from the database based on alphabetical search. Retrospective studies of unidentified data of deceased patients who did not decline research participation are generally allowed by our institution without an additional approval procedure. All the autopsies and microscopic confirmations of PC diagnosis were conducted by experienced pathologists. Presence of DM, length of its duration, and its duration prior to the diagnosis of PC, as well as symptoms leading to the investigation, were based on data from the medical records. Chi-square analyses were conducted to determine whether frequency of DM diagnosis differed significantly between PC and control groups. In addition, a subanalysis based on sex status of the persons was conducted.

## 3. Results

The data of 182 patients (84 males, 98 females) who died due to PC and 135 controls (77 males, 58 females) were analyzed. The mean age of the patient population was 68.7 ± 10.7 years (67.6 for males, 69.6 for females) and 71.0 ± 11.7 years for the controls (69.2 for males, 71.7 for females). The difference was statistically insignificant (*P* = 0.066). The youngest enrolled person was 34 years old, the oldest was 96.

The clinical symptoms leading to medical investigation and PC diagnosis, including their length, are summarized in Table 1. The symptoms preceded PC on average only by weeks to months.

The causes of death from medical documentation of patients in the control group are summarized in Table 2. 

DM was present in 64 PC patients (35.2%); 21 were male (25% of male population) and 43 were female (43.9% of female population). Therefore, the prevalence of DM was significantly higher among female PC patients (*χ* ^2^ = 7.070; *P* = 0.008). 

DM was present in 27 patients (20.0%) in the control group; 17 were male (22.1%) and 10 were female (17.2%). Therefore, the significant difference in DM prevalence among the sexes seen in the PC patient group was not found in the control group (*χ* ^2^ = 0.484; *P* = 0.487).

In global, chi-square analyses revealed a statistically significant difference in the prevalence of DM among patients comprising the PC and control group (*χ* ^2^ = 8.709; *P* = 0.003). 

The average duration of DM prior to the diagnosis of PC was 8.2 years (range of 1 to 23 years). In 18 PC patients (28.1% of the PC group; 8 male, 10 female), DM was diagnosed less than 3 years prior to PC diagnosis. In regard to sex differences, DM was a new-onset diagnosis in 23.3% of diabetic females compared to 38.1% new-onset male diabetics (*χ* ^2^ = 1.537; *P* = 0.215). Thus, the trend in the difference in onset of DM among the sexes in the PC group did not reach statistical significance, partially because of small sample size. The results are summarized in Table 3.

## 4. Discussion

PC is a disease with a dismal prognosis that is characterized by a typically long, asymptomatic course and with limited detection of early curative stages [14]. The majority of patients (>85%) have unresectable disease by the time disease-specific symptoms develop and the diagnosis is made [4]. Correspondingly, the symptoms in our PC patients preceded the PC diagnosis on average only by weeks to months (Table 1). Therefore, in order to detect surgically treatable stages of PC, asymptomatic individuals must be screened [13].

The incidence of PC is too low for cost-effective screening within the general population. For effective and economical screening, it is necessary to establish risk factors and screen persons at high risk for PC development [13]. Currently, there are screening programs for patients with several genetic disorders, who are at higher risk for PC progression [5, 15–19]. However, high risk groups in the general population have not yet been well defined [20]. 

Diabetes mellitus is a factor that has long been discussed in relation to PC. While a meta-analysis published in 2011 confirmed the overall increased risk of PC among diabetics (RR = 1.94), subgroup analysis revealed that the relative risk of PC was correlated negatively with the duration of DM, with the highest risk of PC found among patients diagnosed with DM within 1 year (RRs = 5.38) [21]. It is reasonably believed that in some individuals a new-onset DM may be the first symptom of an otherwise asymptomatic PC [10, 11, 22, 23].

The prevalence of DM among PC patients in this study was 35.2%. In comparison, diabetes was present in 20.0% of patients in the control group. This difference was statistically significant, and it supports the role of DM in PC. These results are consistent with other reports in the literature, which have documented rates of DM in PC patients ranging from 8.5%–40% [10, 11, 22, 23]. 

Our results (DM in PC patients 35.2% versus controls 20.0%) are surprisingly similar to the results of a recent study by Chari et al. [23], who found DM in 40.2% of patients and in 19.2% of controls using fasting blood glucose levels and/or antidiabetic medication for DM identification. They report that prevalence of DM was similar in PC patients and controls 3 years before PC diagnosis. A continuous increase of DM prevalence was observed as time approached PC diagnosis in the patient group, while it remained stable within the control group. 

The prevalence rate reported in this study is unusually high for retrospective autopsy methodologies relying on medical records; such studies typically report DM in less than 20% of PC patients [12, 24]. This may be because more than 25% of DM remains undiagnosed and does not enter medical records [25, 26]. In the case of PC, the prevalence of undiagnosed diabetes is believed to be even higher (approximately 50%) because the PC manifests before the DM can become symptomatic and diagnosed [11]. While this may be the situation in a health care system based on personal freedom, such as those in the United States, we believe that the system of annual preventive medical check-ups that used to be organized and enforced by law in the Czech Republic may lead to better detection of conditions like DM in today's generations of seniors.

In cohort studies, the prevalence of DM in the general population over 60 years of age has been reported as 21% in Poland [27] and 16.9% in the USA [28], both of which are comparable with the prevalence of 20.0% in our control group. This lends support to the accuracy of our retrospective results when compared with prospective studies. 

DM was more common in female PC patients than in male PC patients (43.9% and 17.2%, respectively) and a difference of this magnitude was not seen among the control group patients. To our knowledge, no study thus far has compared DM prevalence among the sexes in patient and control groups. The sex status based comparison of DM prevalence among PC patients alone has been evaluated by several studies. In agreement with our results, Souza et al. found a higher prevalence of DM among female patients in their retrospective study of 151 PC patients [29]. The reason for this difference is unclear, but according to this study, it cannot be explained by higher BMI in women, as BMI was comparable among the sexes. However, Pannala's study provided opposite results, with males exhibiting DM more frequently than females [22]. Additionally, other studies did not find an increased risk of DM associated with PC in women versus men [30]. However, female diabetics have been suggested to have a higher risk of PC development [6]. This issue deserves further research, as a sex status might represent an additional risk factor useful for diabetics risk stratification with long-standing female diabetics and new-onset male diabetics being at a higher risk of PC developement.

New-onset DM (less than 3 years prior to PC) was diagnosed in 28.1% of our PC patients with diabetes. The percentage of patients with differently defined new-onset diabetes is reported to be 52–100% of all the diabetic PC patients [11, 31]. Our data did not suggest such a high prevalence, which may be partially explained by our retrospective methodology; in our opinion, a retrospective method may prove more reliable in detecting total numbers than detail temporal associations [32]. Additionally, the heterogeneity of new-onset DM definitions in the published literature often makes the comparison difficult.

Patients with newly diagnosed DM are at a substantially increased risk of PC appearnce during the first few years of followup [10, 11, 33]. In the majority of the studies, including the current study, the patients diagnosed with DM did not present with any other symptoms of PC; therefore, DM may be considered the first symptom of the cancer [23]. Our study was not capable of unequivocally verifying this hypothesis, but the short duration of symptoms leading to diagnosis of advanced PC compared to duration of DM is suggestive. Moreover, PC appears to be resectable in most of the patients at the time of DM onset and therefore, this situation might represent a valuable tool in screening for PC [34, 35].

However, PC diagnosed within 3 years of the DM diagnosis represents only about 1% of newly diagnosed diabetics over 50 years of age [11]. Thus, not even this group can be considered a high risk group and tested with sophisticated modern diagnostic methods. Therefore, researchers have worked on criteria distinguishing between DM caused by PC and “common” type 2 DM [11, 12, 32]. Several studies have screened patients with new-onset DM and defined clinical symptoms resulting in frequent diagnosis of PC [12, 36]. Unfortunately, the resectability rates were low, likely because clinical PC symptoms were used to identify the subjects for screening and the short duration of cancer related symptoms prior to the diagnosis of advanced PC was demonstrated by us as well as by others [23]. Pannala et al. conclude, that before the onset of PC symptoms, the clinical profile of PC-associated DM is not very different from that of the patient with type 2 DM and does not help to distinguish between these two forms of DM [22]. Further research is necessary to clarify this controversial issue.

The differentiation between the two types of DM for PC would be simplified by assessment of putative factors produced specifically by PC cells, but their clinical use is usually disappointing [37]. A screening of patients with the combination of new-onset DM and positive family history of PC seems to be useful for detecting of early or premalignant changes [38].

Our study's main limitations include its retrospective nature and inability to conclusively answer epidemiological questions due to a patient group versus control group comparison design. However, the prevalence of pancreatic cancer in the population of the Czech Republic was previously established by our research group as 19.1 per 100,000 males and 18.2 per 100,000 females in 2007. In the time period of 1989–2005, the prevalence of pancreatic cancer increased by 45.9% in males and by 119.1% in females [39, 40]. Similarly, our research was not focused on determining the percentage of clinically undiagnosed PC among autopsied patients. 

Our study's strengths include the data comparison of well-documented groups in which the diagnoses were verified by autopsy. Our study contributes a new perspective with findings of a significantly higher prevalence of DM among female PC patients while DM prevalence was equally represented among the sexes in the control group. The reason for this is not fully understood and this study suggests that there is a potential for future research in this area. Additionally, the verification of the data gathered mostly on the Northern American continent on the population of the Czech Republic is valuable because it decreases the risk of population selection bias.

## 5. Conclusions

As a first-of-its-kind study from Central/Eastern Europe, we provided results of a high prevalence of DM among PC patients which was new-onset in a significant portion of patients. DM was predominant in females among the PC patients, while the DM prevalence was similar among sexes in the control group. The reason for this is unknown and deserves further research, as it might be useful for risk stratification. In general, patients with new-onset DM, especially those presenting with a positive family history of PC and/or atypical symptoms (rapid progression toward insulinotherapy, instability, and weight loss despite intensive treatment, recurrent infections including mycotic, additional abnormalities in laboratory values, and/or on abdominal sonography), should be investigated with highly sensitive imaging methods, including the preferred use of endosonography, to exclude asymptomatic pancreatic malignancy. There is an urgent need for a biomarker identification that would facilitate the definition of high-risk individuals among newly diagnosed diabetics. This practice could lead to the diagnosis of earlier stages of the disease, allowing the curative surgery and more favorable prognosis. Prospective studies are necessary to verify the potential of high risk groups defined this way and the implications of their screening on morbidity and mortality. 

## Figures and Tables

**Table 1:** Symptoms preceding the diagnosis of PC.

Symptoms leading to investigation	Present in number of patients (%)	Average length prior PC diagnosis	Shortest and longest interval
Weight loss	75 (41.2%)	3.1 month	1 week–1 year
Painless icterus	50 (27.5%)	1.5 week	2 days–2 month
Back or abdominal pain	44 (24.2%)	2.2 month	1 week–1 year
Dyspepsia, loss of appetite, nausea, vomiting	41 (22.5%)	2.6 month	1 week–1 year
Ascites	14 (7.7%)	2.3 week	1 week–1 month

**Table 2:** Causes of death of control group patients.

Cause of death according to medical records	Number of patients (%)
Stroke, intracerebral hemorrhage	18 (13.4%)
Cardiovascular disease	52 (38.8%)
Cancer (not PC)	31(23.1%)
Terminal bronchopneumonia	21 (15.7%)
Other	12 (9.0%)

**Table 3:** DM in PC patients and control group.

	PC patients	Control group	
Population (M/F)	182 (84/98)	135 (77/58)	
Mean age (years) (M/F)	68.7 ± 10.7 (67.6/69.6)	71.0 ± 11.7 (69.2/71.7)	*P* = 0.066
DM	64 (35.2%)	27 (20.0%)	*χ* ^ 2^ = 8.709; *P* = 0.003
DM (M/F)	21 (25%)/43 (43.9%)	17 (22.1%)/10 (17.2%)	
	*χ* ^ 2^ = 7.070; *P* = 0.008	*χ* ^ 2^ = 0.484; *P* = 0.487
New-onset DM (M/F)^∗^	8 (38.1%)/10 (23.3%)	3 (17.6%)/2 (20%)	
	*χ* ^ 2^ = 1.537; *P* = 0.215	*χ* ^ 2^ = 0.023; *P* = 0.879

^∗^DM diagnosed less than 3 years prior to PC diagnosis.
